# A pan-cancer analysis of the FAT1 in human tumors

**DOI:** 10.1038/s41598-022-26008-1

**Published:** 2022-12-14

**Authors:** Ziyao Wang, Kang Lin, Hai Xiao

**Affiliations:** 1 The First Clinical Medical College of Gannan Medical University, Ganzhou, 341000 Jiangxi China; 2 Tongji University School of Medicine, Shanghai, 200090 China

**Keywords:** Cancer, Immunology, Biomarkers

## Abstract

FAT atypical cadherin 1 (FAT1) is one of the most mutagenic genes in tumors, and several critical studies have revealed its role in tumors, although no pan-cancer studies are currently available. Therefore, we explored the potential oncogenic role of FAT1 in 33 tumors based on The Cancer Genome Atlas and Gene Expression Omibus datasets. We found that FAT1 was strongly expressed in most tumors and significantly correlated with their prognosis. Additionally, we analyzed the association of FAT1 with tumors from multiple perspectives, including single-cell sequencing, mutations, high tumor mutational burden, microsatellite instability, immune cell infiltration, and immune microenvironment. Our first pan-cancer study provided a relatively comprehensive understanding of the oncogenic role of FAT1 in tumors.

## Introduction

The recent article published in *Nature*^1^ revealed that FAT atypical cadherin 1 (FAT1) mutations play critical roles in tumors and, combined with a literature analysis, highlighted its multiple functions in different tumors. At this stage, performing pan-cancer analysis of significant genes in combination with high-volume tumor sequencing data was crucial and feasible.

The public database, TCGA project^2,3^, and GEO database^4^, containing functional genomic datasets of different tumors, enabled us to perform a pan-cancer analysis. FAT1 encodes a protocadherin, which is one of the most frequently mutated genes in human cancers and plays various functions^1,5,6^. However, there was no evidence of its relationship with multiple tumor types.

Our study was the first pan-cancer analysis of FAT1 using the TCGA and GEO databases. We explored the potential molecular mechanisms of FAT1 in different tumorigenesis and clinical prognosis in terms of gene expression, survival status, genetic alterations, protein phosphorylation, immune infiltration, associated cellular pathways, and marker gene relevance.

## Materials and methods

### Study design

Our study was deemed exempt from institutional board approval and patient informed consent because all data were deidentified and publicly available. This study analyzed the multiple features of FAT1 in the cancer genome atlas pan-cancer cohort to pave the road for in-depth experimental studies. We confirmed that all methods were carried out following relevant guidelines and regulations.

### Data and tools

The TCGA (https://portal.gdc.cancer.gov/) database contained sequencing and clinicopathological data from patients with over 30 different types of tumors. Sequencing data of 33 tumors were downloaded from the TCGA database (10,327 tumor samples and 730 matched paraneoplastic samples) (Table 1).

**Table 1:** Pan-cancer samples in TCGA database and tumor abbreviations.

Tumor abbreviation	Disease Type	TCGA-samples	Normal	Tumor	Expression in tumor
ACC	Adrenocortical carcinoma	79	0	79	↑*
BLCA	Bladder Urothelial Carcinoma	430	19	411	–
BRCA	Breast invasive carcinoma	1217	113	1104	↓***
CESC	Cervical squamous cell carcinoma and endocervical adenocarcinoma	309	3	306	↑*
CHOL	Cholangiocarcinoma	45	9	36	↑***
COAD	Colon adenocarcinoma	512	41	471	↑***
DLBC	Lymphoid Neoplasm Diffuse Large B-cell Lymphoma	48	0	48	↑*
ESCA	Esophageal carcinoma	173	11	162	↑**
GBM	Glioblastoma multiforme	173	5	168	↑*
HNSC	Head and Neck squamous cell carcinoma	546	44	502	↑***
KICH	Kidney Chromophobe	89	24	65	↓***
KIRC	Kidney renal clear cell carcinoma	607	72	535	↓***
KIRP	Kidney renal papillary cell carcinoma	321	32	289	↑***
LAML	Acute Myeloid Leukemia	151	0	151	↓*
LGG	Brain Lower Grade Glioma	529	0	529	↑*
LIHC	Liver hepatocellular carcinoma	424	50	374	↑***
LUAD	Lung adenocarcinoma	585	59	526	↑***
LUSC	Lung squamous cell carcinoma	550	49	501	↑***
MESO	Mesothelioma	86	0	86	–
OV	Ovarian serous cystadenocarcinoma	379	0	379	–
PAAD	Pancreatic adenocarcinoma	182	4	178	↑*
PCPG	Pheochromocytoma and Paraganglioma	186	3	183	–
PRAD	Prostate adenocarcinoma	551	52	499	–
READ	Rectum adenocarcinoma	177	10	167	–
SARC	Sarcoma	265	2	263	–
SKCM	Skin Cutaneous Melanoma	472	1	471	–
STAD	Stomach adenocarcinoma	407	32	375	↑***
TGCT	Testicular Germ Cell Tumors	156	0	156	–
THCA	Thyroid carcinoma	568	58	510	↓***
THYM	Thymoma	121	2	119	–
UCEC	Uterine Corpus Endometrial Carcinoma	583	35	548	–
UCS	Uterine Carcinosarcoma	56	0	56	–
UVM	Uveal Melanoma	80	0	80	–
In total		11,057	730	10,327	20

↑ high expression; ↓ low expression; *P < 0.05; **P < 0.01; ***P < 0.001.

For some tumors without normal or highly restricted normal tissues, their expression data from the GTEx database were obtained using GEPIA2 (http://gepia2.cancer-pku.cn/) webserver^7^. Additionally, survival duration information of 10,327 tumor samples was also downloaded from the TCGA database (including OS, DSS, DFI, and PFI). The gene mutation data of 10,114 pan-cancer samples and the MSI score information of 10,415 tumor samples were downloaded from the TCGA database. The pan-cancer samples and analytical tools included in Sangerbox 3.0 were used in this study. This study mainly used R version 3.6.1 software (https://www.r-project.org/) and various practical packages to process and visualize the data. Other web servers used are introduced separately in different sections throughout the manuscript. All data used in this study were standardly cited as per the requirements of public databases and were permitted for use.

### Differential expression analysis

The mRNA differential expression of FAT1 in pan-cancer was analyzed using the ggpubr package and Wilcox test. For tumors lacking paracancerous samples and insufficient sample size (ACC, CESC, DLBC, ESCA, GBM, LAML, LGG, OV, PAAD, SKCM, TGCT, and UCS), the differential expression box map of FAT1 in “Match TCGA normal and GTEx data” was searched in GEPIA2, with p-value Cutoff = 0.01, |Log2FC|Cutoff = 1. To confirm the reliability of our results, the network tool Sangerbox 3.0 (http://vip.sangerbox.com/) database was used to search the expression of FAT1 in pan-cancer. This tool is based on the unified and standardized pan-cancer data set of UCSC Genome Browser (http://genome-asia.ucsc.edu/): TCGA TARGET GTEx (PANCAN, N = 19,131, G = 60,499). The expression data of FAT1 were extracted, the samples with zero expression levels were filtered, and the expression values were converted using log2 (x + 0.001). The cancer species with less than three samples in a single cancer specie were excluded, and the difference was analyzed by unpaired Wilcoxon Rank Sum and Signed Rank Tests. Oncomine^8^ (https://www.oncomine.org/) tumor data set was also included to analyze the mRNA expression of FAT1. We combined data of FAT1 expression and tumor staging of patients, used the Wilcox test of the limma package for analysis, controlled the false discovery rate (FDR) below 0.05, and then plotted the graphs using ggpubr. We also searched the protein expression of FAT1, immunohistochemistry^9^, and single-cell RNA expression in UALCAN^10^ (http://ualcan.path.uab.edu/) and The Human Protein Atlas (https://www.proteinatlas.org/).

### Survival prognosis analysis

We searched the relationship between FAT1 expression and pan-cancer patients prognosis using Sangerbox, which is based on the TCGA TARGET GTEx dataset. The TCGA prognosis study published in *Cell* journal^11^ provided a high-quality prognostic data set of TCGA. TARGET follow-up data were obtained from UCSC as supplementary data. Additionally, the samples with no expression level and follow-up duration of less than 30 days were excluded, and the expression data were transformed using log2 (x + 0.001). Additionally, the cancers with less than ten samples were also excluded, and the pan-cancer expression data and the OS, DSS, DFI, and PFI data of the corresponding samples were also retrieved. Cox proportional hazard regression model was established by the coxph function of survival (version 3.2–7) package to analyze the relationship between gene expression and prognosis in each tumor. The Kaplan–Meier method was used to determine the prognostic value of FAT1 based on the downloaded transcriptome and clinicopathological data. Log-rank test was used to obtain a significant prognosis.

### Mutation analysis

The cBioPortal database (http://www.cbioportal.org/)^12^ is a web service that integrates the downloading of tumor datasets with online analysis and provides a variety of robust and reliable analysis tools. The mutations of FAT1 in pan-cancer (including mutations, structural variants, and copy number alterations) were analyzed based on the 32 studies selected (10,967 samples, TCGA, PanCancer Atlas). Additionally, all likely oncogenic mutations (OncoKB), Post Translational Modifications (PTMs), mutated exons, and details regarding the subcellular location of the mature FAT1 protein were analyzed. Gene mutations may affect the survival of tumor patients. To analyze the relationship between FAT1 mutations and patient prognosis, the first four tumors (HNSC, UCEC, LUSC, SKCM) with more than 15% mutation rates were selected based on the survival module of the database. The clinical response of ICIs can be predicted by TMB. The mutations of FAT1 and its function in predicting patient prognosis were analyzed using the 1661 patients/samples of TMB and immunotherapy (MSKCC, Nat Genet 2019^13^) dataset.

### TMB and MSI

We retrieved the gene mutation data of 10,114 pan-cancer samples from the TCGA database to calculate the mutation score of each sample and obtained tumor mutation burden. The correlation between the expression of FAT1 and TMB was analyzed using Spearman’s correlation test, and the correlation radar chart was drawn using the fmsb package. The MSI scores of 10,415 tumor samples were downloaded and analyzed, resulting in a radar map of the MSI correlation between FAT1 and tumors.

### TME and immune cell infiltration

Based on the TCGA Pan-Cancer (PANCAN, N = 10,535, G = 60,499) dataset in Sangerbox, the samples with zero expression levels were filtered, and the expression values were transformed by log2 (x = 0.001). The stromal, immune, and ESTIMATE scores for each patient in each tumor were calculated using the R software package ESTIMATE^14^ (https://bioinformatics.mdanderson.org/estimate/), according to gene expression. The immune infiltration scores of 9530 tumor samples from 39 tumor types were obtained. The Spearman’s correlation coefficient of FAT1 expression and TME was calculated based on the psych package’s corr.test function, which helped determine the significant correlation between immune infiltration scores. Similarly, samples with zero expression levels were filtered based on the TCGA TARGET GTEx (PANCAN, N = 19,131, G = 60,499) dataset in Sangerbox, and the expression values were transformed by log2 (x = 0.001). Each patient’s immune cell infiltration score in each tumor was evaluated using the deconvo_CIBERSOR method of the IOBR package^15,16^, following gene expression. Finally, 22 immune cell infiltration scores were obtained from the 10,063 tumor samples of 44 tumor types. The Spearman’s correlation coefficient of FAT1 expression and immune cell infiltration score in each tumor was also calculated using the psych package’s corr.test function to determine the significantly related immune infiltration score.

### Protein expression and protein–protein interaction (PPI)

The Human Protein Atlas (https://www.proteinatlas.org/) database was searched for information about FAT1 protein expression in 17 different types of tumor tissues and paracancerous tissues that had been validated by immunohistochemistry. We also explored the expression of FAT1 in different kinds of single cells. STRING (https://string-db.org/)17 is a known and predicted PPI database that was used to analyze the PPI network of FAT1 in this study. The cytoHubba plug-in of Cytoscape was used to analyze the degree of each protein, and then the PPI network was drawn. We also searched the pathways and genes that interact with FAT1 in UCSC Genome Browser. The Gene Ontology and KEGG Pathway were analyzed in Metascape (https://metascape.org/) based on the interacting proteins in the PPI network.

### Protein phosphorylation

PhosphoNET (http://www.phosphonet.ca/) is the world’s largest online repository of general and predictive information about human phosphorylation sites, their evolutionary conservation, the identity of protein kinases that may target these sites, and related phosphate sites. The verified and predicted phosphorylation sites were obtained by searching FAT1. The most significant phosphorylation sites and kinases involved in phosphorylation were predicted by calculating the Kinase Predictor V2 Score by the Kinexus P-Site Prediction algorithm.

### Correlation analysis of marker genes

Studies have confirmed that various biological processes are involved in the occurrence and development of malignant tumors. Through the review of the literature, we found that FAT1 can participate in multiple functions. The literature review also helped identify the gene markers in EMT^18^, tumor hypoxia^19^, exosome^20^, tumor immunity^21^, methylation^22,23^, and autophagy^24,25^. The correlation between the mRNA expression levels of FAT1 and these marker genes in pan-cancer was obtained by Spearman correlation analysis with the limma package. The results can predict the potential benefits of FAT1 in these processes and lay a bioinformatics foundation for further studying FAT1 in malignant tumors.

### Data analysis

Wilcox and T-tests were used for FAT1 differential expression analysis. Cox proportional hazard regression model and Kaplan–Meier methods were used for survival analysis, and Spearman’s analysis was used for studying correlations. Except for special instructions, *P* < 0.05 demonstrated that the difference was statistically significant. *P < 0.05. **P < 0.01. ***P < 0.001.

## Results

### FAT1 expression

**Figure 1:**
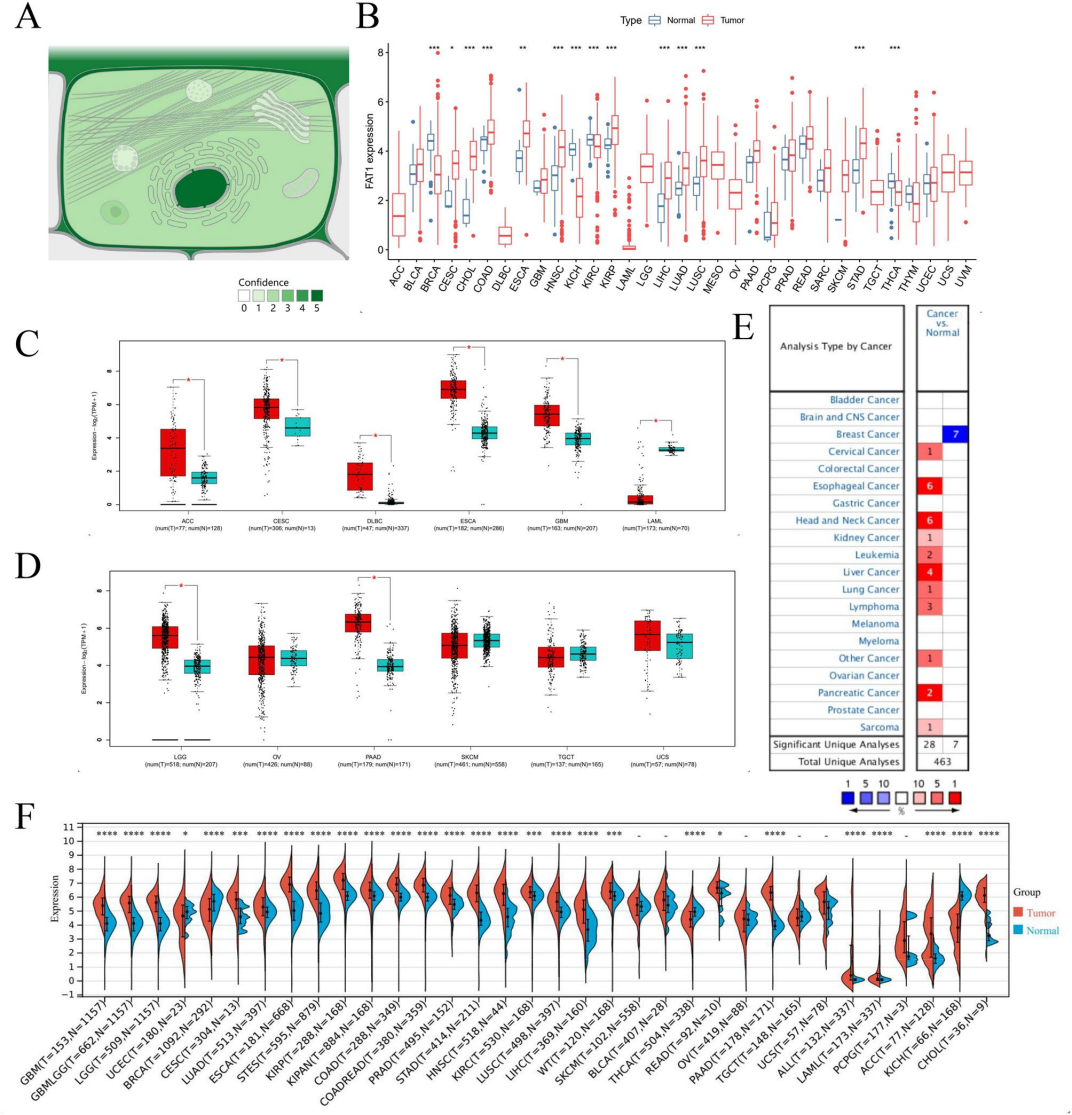
Subcellular locations and mRNA expression levels of FAT1. (**A**) Subcellular locations of FAT1 based on protein expression. (**B**) mRNA expression levels of FAT1 in pan-cancer. (**C**, **D**) mRNA expression levels of FAT1 in 12 tumors. (**E**) FAT1 expression levels in the dataset of 20 tumors in the Oncomine database. The best gene rank percentile determines the cell color for the analyses within the cell. The red indicates high expression, and the blue indicates low expression. (**F**) mRNA expression of FAT1 in pan-cancer from the Sangerbox database.

**Figure 2:**
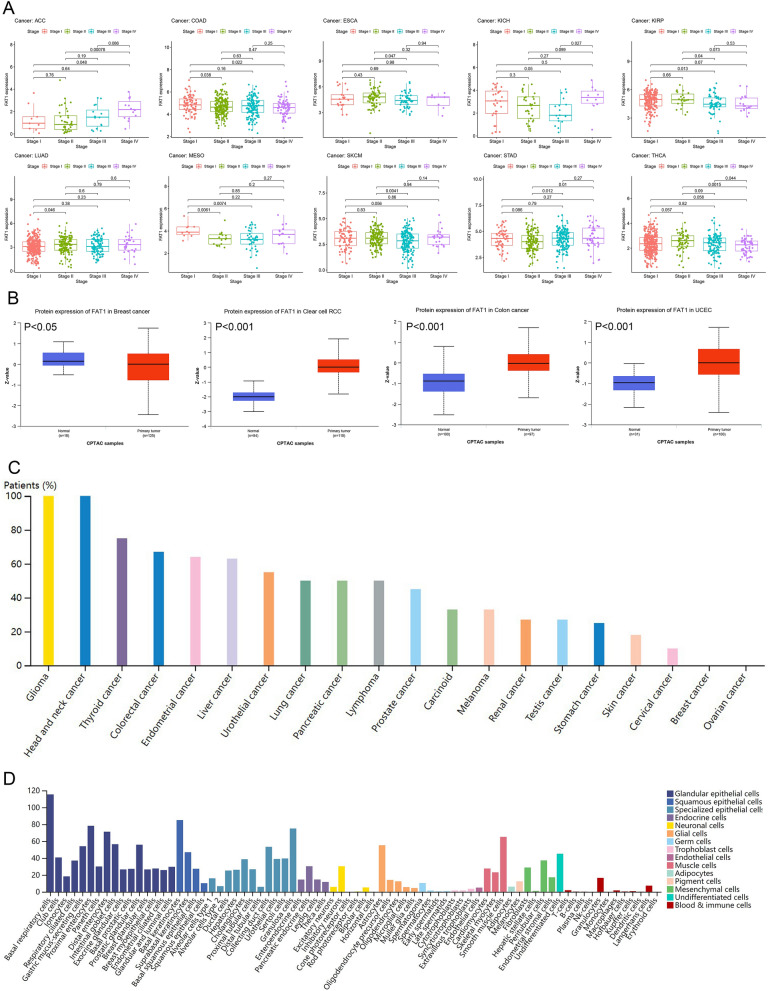
FAT1 multi-omics expression. (**A**) FAT1 mRNA levels in pan-cancer with differential expression at different stages. (**B**) Based on CPTAC data, FAT1 protein is expressed at low levels in breast cancer and highly expressed in clear cell renal cell carcinoma, colon cancer, and uterine corpus endometrial carcinoma. (**C**) Immunohistochemical summary histogram of FAT1 in pan-cancer based on The Human Protein Atlas, different colors represent different tumors. (**D**) Expression levels of FAT1 in 12 single cell types based on The Human Protein Atlas.

According to the subcellular locations from COMPARTMENTS (https://compartments.jensenlab.org/), FAT1 protein is mainly expressed in the nucleus, plasma membrane, and extracellular regions of the cell^26–28^ (Fig. 1A). We found that FAT1 was highly expressed in pan-cancer, except for low expression in some tumors (BRCA, KICH, KIRC, LAML, THCA, UCEC) and no differential expression in some tumors (BLCA, OV, PCPG, SKCM, SARC, TGCT, THYM, UCS) (Fig. 1B–F) based on the computational analysis of FAT1 mRNA expression and multi-database analysis. The analysis of FAT1 expression in different tumor stages revealed its significant difference in various tumors like ACC, COAD, ESCA, KICH, KIRP, LUAD, MESO, SKCM, STAD, and THCA (Fig. 2A). Interestingly, there was no significant difference in FAT1 expression between SKCM tumor and normal tissues. FAT1 protein expression was significantly higher in clear cell renal cell carcinoma, colon cancer, and uterine corpus endometrial carcinoma and was significantly lower in breast cancer. There was no significant difference in FAT1 protein expression in lung adenocarcinoma and ovarian cancer (Fig. 2B). The noteworthy point is that, contrary to our findings, Nantana Kwaepila et al.^29^ reported that FAT1 in breast cancer immunohistological expression data displayed high expression levels in human tumor samples, possibly due to sample specificity issues. High- or medium-intensity staining (Fig. 2C) was observed in the tumor tissues of glioma, head and neck, thyroid, colorectal, endometrial, liver, urothelial, lung, pancreatic cancers, and lymphomas, indicating that the FAT1 protein was significantly overexpressed in the tumor tissues of these diseases. We also found that FAT1 was highly expressed in many glandular epithelial cells, squamous epithelial cells, and specialized epithelial cells based on the summary of single-cell expression of FAT1. It was also highly expressed in astrocytes, smooth muscle cells, and other cells (Fig. 2D).

### Survival analysis

**Figure 3:**
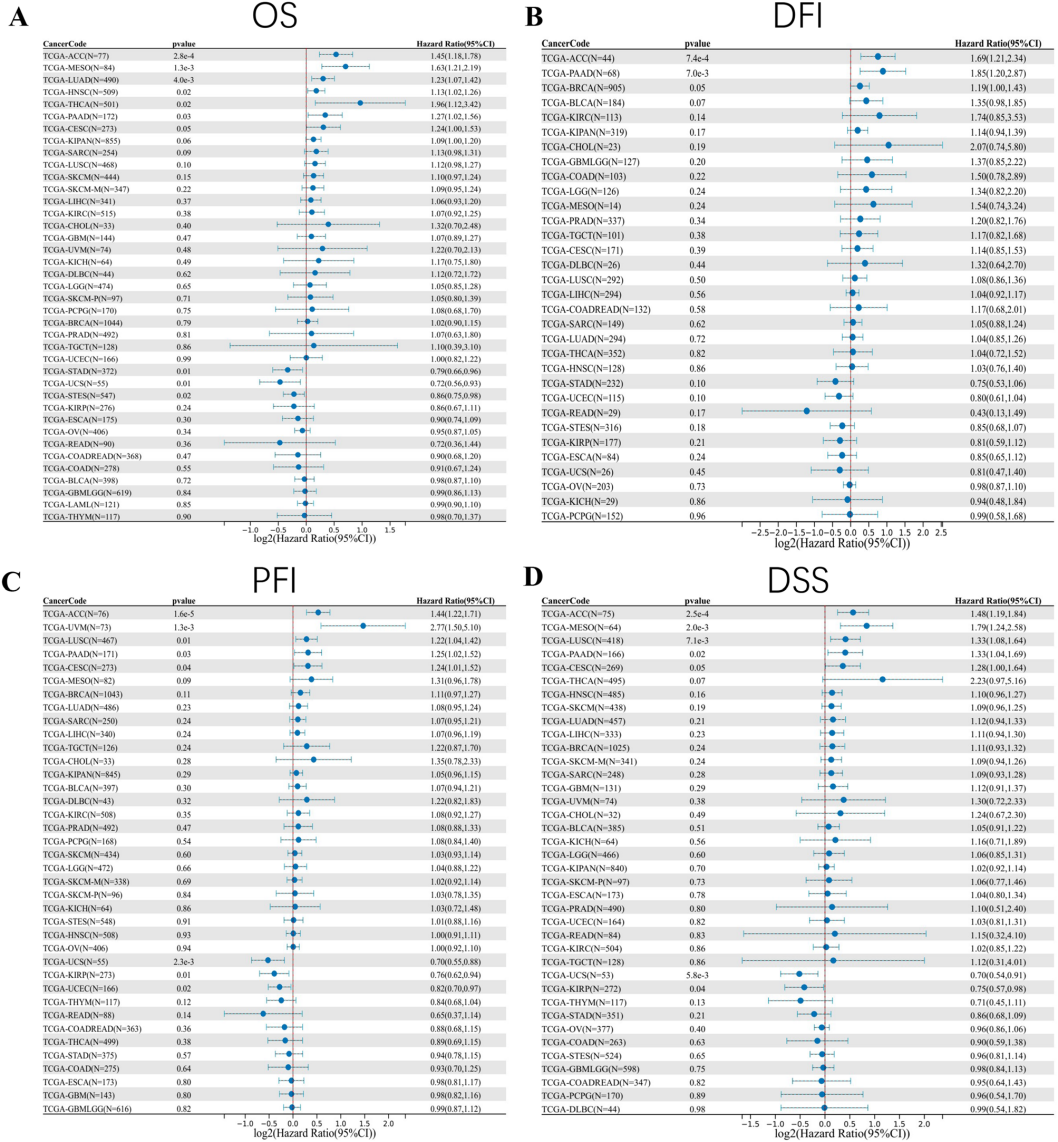
Sangerbox database analysis of the prognostic value of FAT1 mRNA expression in pan-cancer. (**A**) Overall survival. (**B**) Disease-free interval. (**C**) Progression-free interval. (**D**) Disease-specific survival.

Combining the OS, DSS, DFI, and PFI results of FAT1 calculated by the Cox proportional hazard regression model (Fig. 3) and Kaplan–Meier (Fig. S1), we found that the high FAT1 mRNA expression predicted a worse prognosis in patients with ACC, BRCA, BLCA, KIRC, SARC, KIRP, MESO, LUSC, LUAD, HNSC, THCA, THYM, PAAD, CESC, OV, SKCM, and UVM. In comparison, it predicted a better prognosis in STAD, STES, ESCA, KIRP, UCS, UCEC, READ, and TGCT.

### Mutation analysis

**Figure 4:**
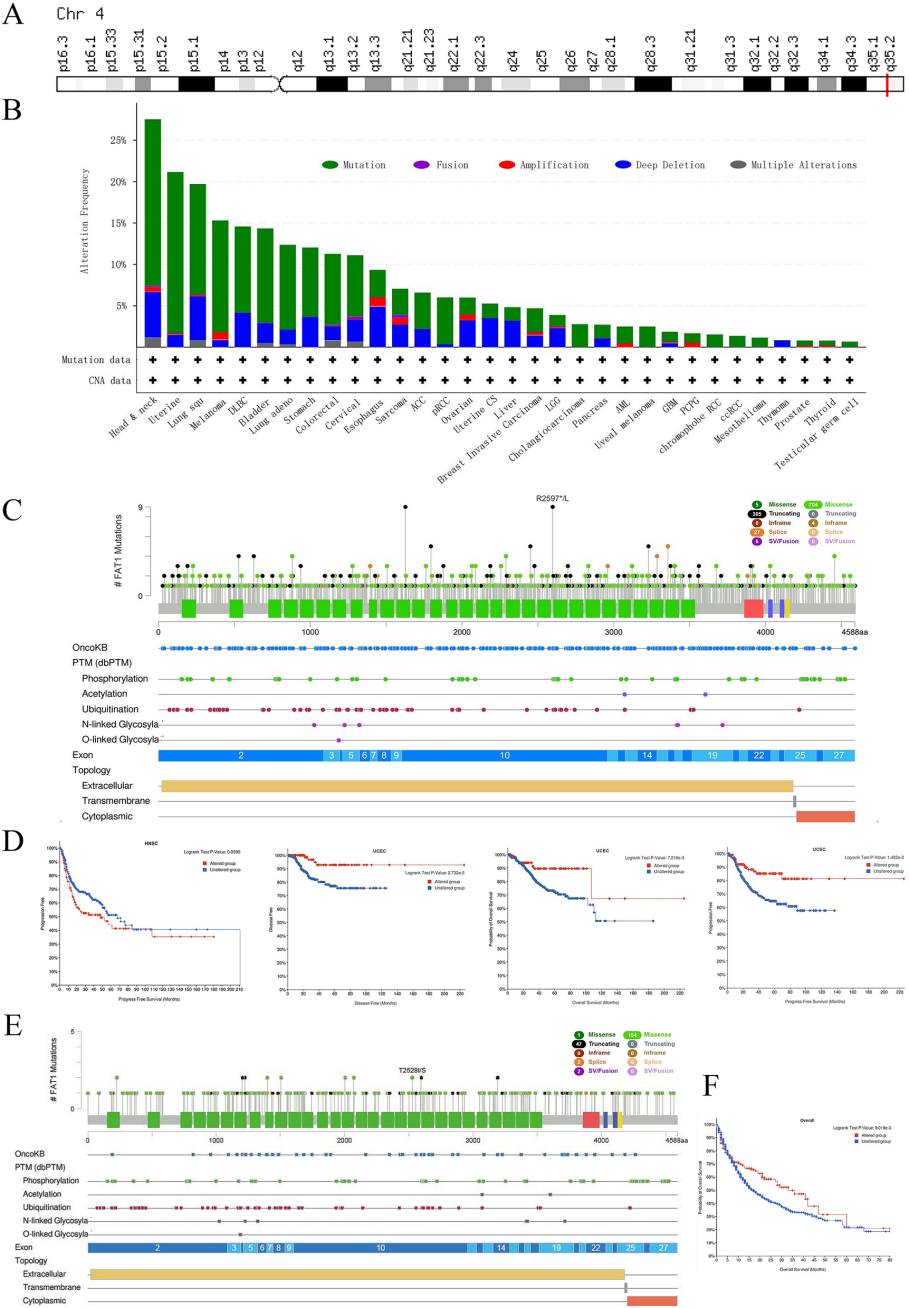
Mutations of FAT1 in pan-cancer. (**A**) Location of FAT1 in the chromosome. (**B**) Mutation of FAT1 in different tumors, “Deep Deletion”, indicates a deep loss, possibly a homozygous deletion, and “Amplification” indicates a high-level amplification (more copies, often focal). (**C**) General overview of FAT1 mutations in pan-cancer, with nodes and annotations representing different mutation profiles. (**D**) FAT1 mutation in UCEC predicts a good prognosis for patients, but in HNSC, it predicts a worse prognosis. (**E**) General overview of FAT mutations in the TMB and immunotherapy (MSKCC, Nat Genet 2019) dataset, with nodes and annotations representing different mutation profiles. (**F**) FAT1 mutations in the TMB and immunotherapy (MSKCC, Nat Genet 2019) dataset are predicted to have a better prognosis.

FAT1 encoding gene is located at q35.2 of chromosome 4 (Fig. 4A). We analyzed the mutational information of FAT1 in pan-cancer using cBioportal and found that FAT1 was mutated in more than 10% of 10 tumors, including HNSC and UCEC (Fig. 4B). Missense and truncating were the two most common mutations of FAT1, which primarily affects extracellular and cytoplasmic regions. The PTMs results depicted that FAT1 proteins are primarily phosphorylated and ubiquitinated (Fig. 4C). The mutation survival analysis of the top four tumors with more than 15% mutation rate demonstrated that FAT1 mutations in UCEC predicted better prognosis for tumor patients, but FAT1 mutations in HNSC predicted poor survival (Fig. 4D). The TMB and immunotherapy dataset revealed that FAT1 mutations were observed in 10% of the samples, which is consistent with the findings from the pan-cancer analysis (Fig. 4E). Notably, the OS indicated that patients with FAT1 mutations had a better prognosis (Fig. 4F), suggesting that FAT1 mutations may be used as an indicator of patient’s prognosis.

### Immunocomprehensive analysis

**Figure 5:**
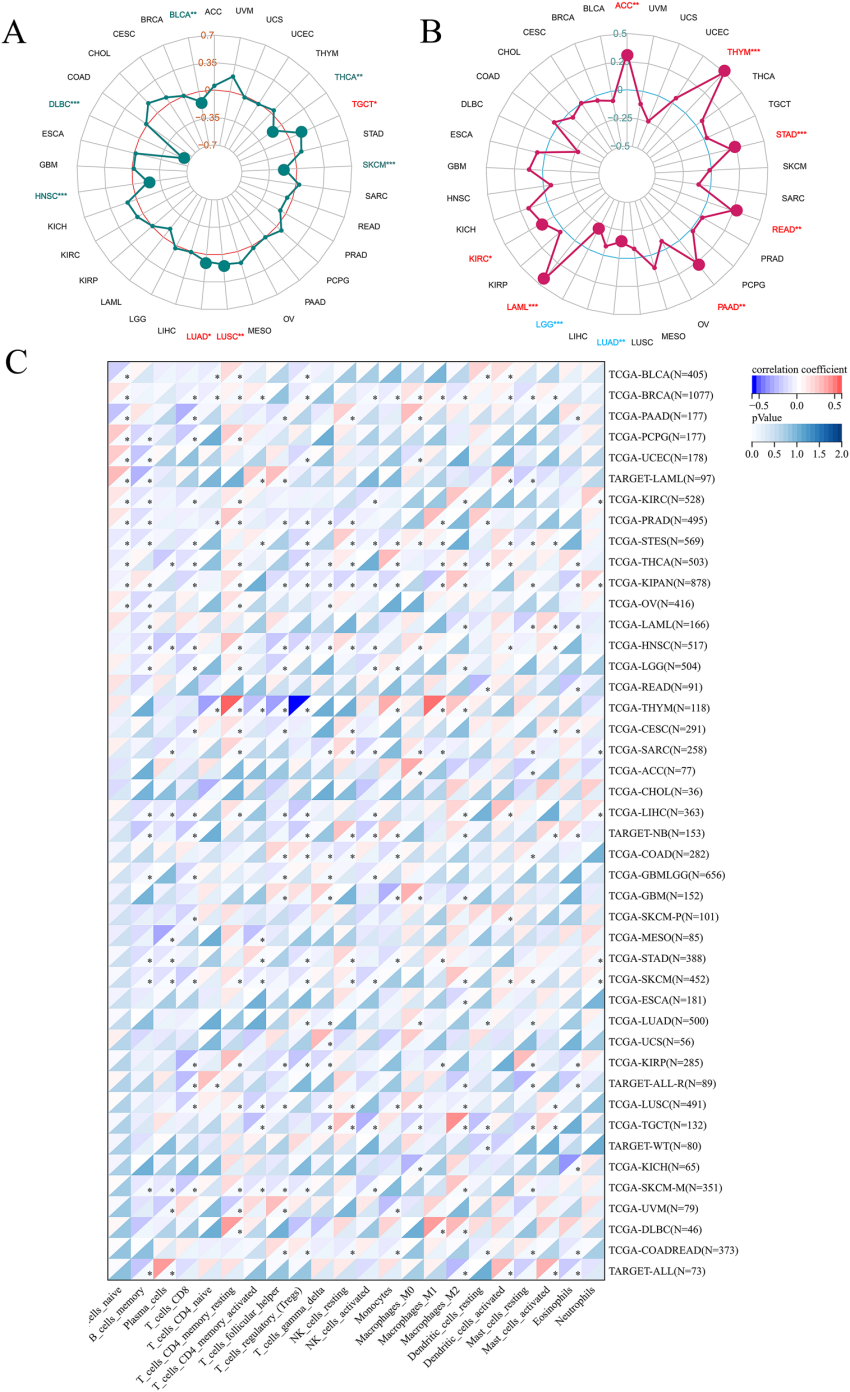
FAT1 correlates with MSI, TMB, and immune in pan-cancer. (**A**) Correlation of FAT1 expression with pan-cancer MSI. (**B**) Correlation between FAT1 expression and pan-cancer TMB. (**C**) Correlation of FAT1 expression with the amount of pan-cancer immune cell infiltration.

Tumor therapy has advanced significantly owing to immunotherapy that targets immunological checkpoints^30^. It has been demonstrated that MSI and TMB^31^ are biomarkers for predicting tumor immunotherapy and are crucial in directing the systemic treatment of tumors^32^. The analysis of sequencing data demonstrated significant correlations between FAT1 and MSI of several tumors, positive correlations in LUAD, LUSC, and TGCT, and negative correlations in BLCA, DLBC, HNSC, SKCM, and THCA (Fig. 5A). Additionally, FAT1 was also significantly correlated with TMB of many tumors; positively correlated in ACC, KIRC, LAML, PAAD, READ, STAD, and THYM, and negatively correlated in LGG and LUAD (Fig. 5B). The TME has a significant role in tumor occurrence and development, as well as in the evaluation of invasion, metastasis, and prognosis, thus pointing out the future direction for more effective immunotherapy^33–35^. Our study confirmed a significant correlation between the FAT1 expression and TME components in various tumors. Analysis was done to explore the correlation between the FAT1 expression and the Estimate score of the matrix score and immune score. The results revealed that FAT1 was positively correlated with BRCA, DLBC, LGG, OV, and PCPG but negatively correlated with GBM, KIRP, PAAD, PRAD, SARC, SKCM, STES, TGCT, THCA, and THYM (Table 2). Immune cells in the TME are essential for tumor regulation^36^. This study analyzed the relationship between FAT1 expression and 22 different types of immune cell infiltration in pan-cancer. The results demonstrated that FAT1 was significantly correlated with one or more immune cell infiltration in all tumor types except CHOL. There was a significant positive correlation with T-cells-CD4-memory-resting and macrophages-M1 of THYM and a significant negative correlation with Tregs (Fig. 5C).

**Table 2:** FAT1 protein phosphorylation.

Phosphosites	P-site Match (Sequence)															Protein Kinase Match (Top 1)			Experimentally confirmed
	-7	-6	-5	-4	-3	-2	-1	0	1	2	3	4	5	6	7	Human Kinase Short Name	Human Kinase Full Name	Kinase Predictor V2 Score	PubMed ID
S150	N	D	L	R	P	L	F	**S**	P	T	S	Y	S	V	S	MAPK8	Mitogen-activated protein kinase 8	593	19,276,368
T152	L	R	P	L	F	S	P	**T**	S	Y	S	V	S	L	P	HIPK1	Homeodomain-interacting protein kinase 1	131	18,220,336
S153	R	P	L	F	S	P	T	**S**	Y	S	V	S	L	P	E	CDK7	Cyclin-dependent kinase 7	387	19,276,368
T192	Y	Y	S	F	K	D	R	**T**	D	M	F	A	I	H	P	CK2a1	Casein kinase II, alpha chain	117	20,068,231
Y212	V	L	T	G	R	L	D	**Y**	L	E	T	K	L	Y	E	CSK	Tyrosine-protein kinase CSK	436	20,068,231
Y400	P	A	Y	S	H	L	R	**Y**	V	F	K	R	T	P	G	SYK	Tyrosine-protein kinase SYK	440	–
T464	N	S	N	P	P	E	F	**T**	Q	T	A	Y	K	A	A	ATR	Serine-protein kinase ATR	378	17,525,332
Y468	P	E	F	T	Q	T	A	**Y**	K	A	A	F	D	E	N	SYK	Tyrosine-protein kinase SYK	357	17,525,332
T771	C	F	M	I	D	M	E	**T**	G	M	L	K	I	L	S	SRC	Proto-oncogene tyrosine-protein kinase Src	103	–
S778	T	G	M	L	K	I	L	**S**	P	L	D	R	E	T	T	p38d	Mitogen-activated protein kinase 13	490	–
T1277	P	L	Y	R	V	I	A	**T**	D	K	D	E	G	P	N	CK2a1	Casein kinase II, alpha chain	115	–
T1306	K	F	F	I	E	P	K	**T**	G	V	V	S	S	K	R	CDK9	Cyclin-dependent kinase 9	125	–
S2621	K	G	T	S	V	V	K	**S**	A	S	D	A	D	E	G	ANPa	Atrial natriuretic peptide receptor A	351	–
T3050	L	I	M	Q	I	S	A	**T**	D	A	D	I	R	S	N	CK2a2	Casein kinase II, alpha' chain	103	20,068,231
S3084	D	T	G	E	L	K	T	**S**	T	P	L	D	R	E	E	NEK4	Serine-threonine-protein kinase Nek4	291	20,068,231
T3404	T	K	L	L	D	R	E	**T**	I	S	G	Y	T	L	T	PIM1	Proto-oncogene serine-threonine-protein kinase Pim-1	202	–
S3406	L	L	D	R	E	T	I	**S**	G	Y	T	L	T	V	Q	SgK307	Testis expressed protein 14	275	–
Y4244	S	K	L	N	K	N	I	**Y**	S	D	I	P	P	Q	V	TEC	Tyrosine-protein kinase Tec	381	18,083,107
S4274	R	N	N	L	D	R	N	**S**	F	E	G	S	A	I	P	PKG1	cGMP-dependent protein kinase 1, alpha isozyme	499	20,068,231
S4278	D	R	N	S	F	E	G	**S**	A	I	P	E	H	P	E	ZC2	TRAF2 and NCK-interacting kinase	327	–
S4316	N	L	P	P	P	P	P	**S**	N	S	P	S	D	S	D	ERK1	Mitogen-activated protein kinase 3	530	–
S4318	P	P	P	P	P	S	N	**S**	P	S	D	S	D	S	I	ERK1	Mitogen-activated protein kinase 3	646	–
S4322	P	S	N	S	P	S	D	**S**	D	S	I	Q	K	P	S	ERK1	Mitogen-activated protein kinase 3	404	–
Y4358	E	E	K	P	S	Q	P	**Y**	S	A	R	E	S	L	S	JAK2	Tyrosine-protein kinase JAK2	320	18,180,459
S4475	R	D	M	P	A	A	G	**S**	L	G	S	S	S	R	N	CaMK2d	Calcium-calmodulin-dependent protein kinase type II delta chain	364	18,578,522
S4478	P	A	A	G	S	L	G	**S**	S	S	R	N	R	Q	R	PIM1	Proto-oncogene serine-threonine-protein kinase Pim-1	348	–
S4479	A	A	G	S	L	G	S	**S**	S	R	N	R	Q	R	F	PKCe	Protein kinase C, epsilon type	384	20,068,231
Y4491	Q	R	F	N	L	N	Q	**Y**	L	P	N	F	Y	P	L	ErbB2	Receptor tyrosine-protein kinase erbB-2	365	18,180,459
Y4521	C	R	E	P	H	A	P	**Y**	P	P	G	Y	Q	R	H	FRK	Fyn-related kinase	360	–
Y4525	H	A	P	Y	P	P	G	**Y**	Q	R	H	F	E	A	P	ZAP70	Tyrosine-protein kinase ZAP-70	372	–

### PPI and functional enrichment analysis

**Figure 6:**
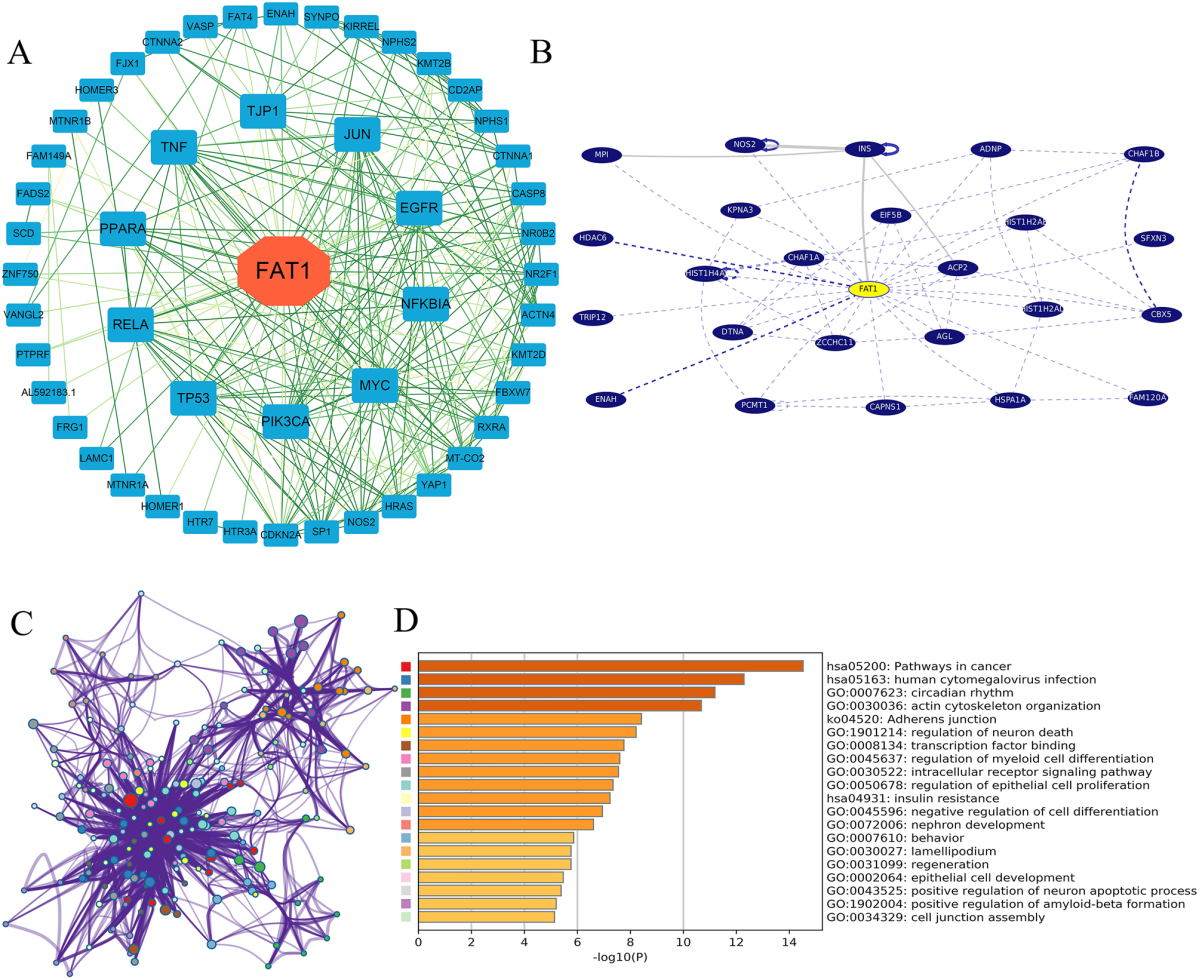
FAT1 protein interaction network and pathway enrichment analysis. (**A**) Protein–protein interaction network of FAT1 in the STRING database, the darker color of the linkage represents stronger correlation, active interaction sources including text mining, experiments, databases, co-expression, neighborhood, gene fusion, co-occurrence, the minimum required interaction score is 0.4 (medium confidengce). (**B**) The top 24 genes with the strongest correlation with FAT1 in UCSC, only FAT1-interacting genes and only the most-mentioned/most-curated interactions, Solid grey lines—only text-mining support for this interaction, with the thickness of the line indicating the number of articles supporting it. Dashed blue lines—at least one curated database supports this interaction. Dark blue—the information is derived from a paper describing fewer than ten interactions. Light blue—the information is derived from a high-throughput paper describing more than ten interactions. Solid blue lines—databases and text mining support this interaction and are colored by support. The top 20 results of KEGG and GO enrichment analysis of FAT1 and its interacting proteins, (**C**) cluster network plot, (**D**) histogram, different colors represent different pathways and biological processes (The color of panel C corresponds to the color in panel D), “Log10 (P)” is the p-value in log base 10.

PPI is significant in a tumor’s molecular biological regulation because of its versatility, specificity, and adaptability^17,37^. The PPI results based on Cytoscape analysis demonstrated that there was a significant interaction between MYC, EGFR, PIK3CA, TP53, TNF, RELA, NFKBIA, TJP1, JUN, PPARA, and FAT1 (Fig. 6A), and the degree of these interacting proteins in the network was more than 15. Second, PPI results from the UCSC tool depicted that FAT1 interacts with many transcription factors (ADNP, CBX5) and essential proteins such as EIF5B histones, AGL, and HSPA1A (Fig. 6B). The results of Gene Ontology and KEGG Pathway enrichment analysis (https://www.kegg.jp/kegg/kegg1.html)^38^ based on the proteins in the PPI network revealed that it was significantly enriched in cancer pathways and particularly related to transcription factor binding, intracellular receptor signaling pathway, regulation of epithelial cell proliferation, negative regulation of cell differentiation, epithelial cell development, and other pathways (Fig. 6C,D).

### FAT1 protein phosphorylation

Protein phosphorylation is recognized as the primary method of protein PTMs, and it is an effective way to regulate protein function under the catalysis of protein kinases. It is a fundamental cause of intracellular processes such as cell growth and development, signal transduction, and metabolism^39,40^. A database search revealed that 30 FAT1 proteins had been phosphorylated (Table 2). Second, data scoring predicted 45 sites most likely to be phosphorylated, and the protein kinases (Table S1) most likely to participate in the phosphorylation process were also predicted. The phosphorylation of the FAT1 protein is now better understood.

### Correlation analysis of tumor marker genes

**Figure 7:**
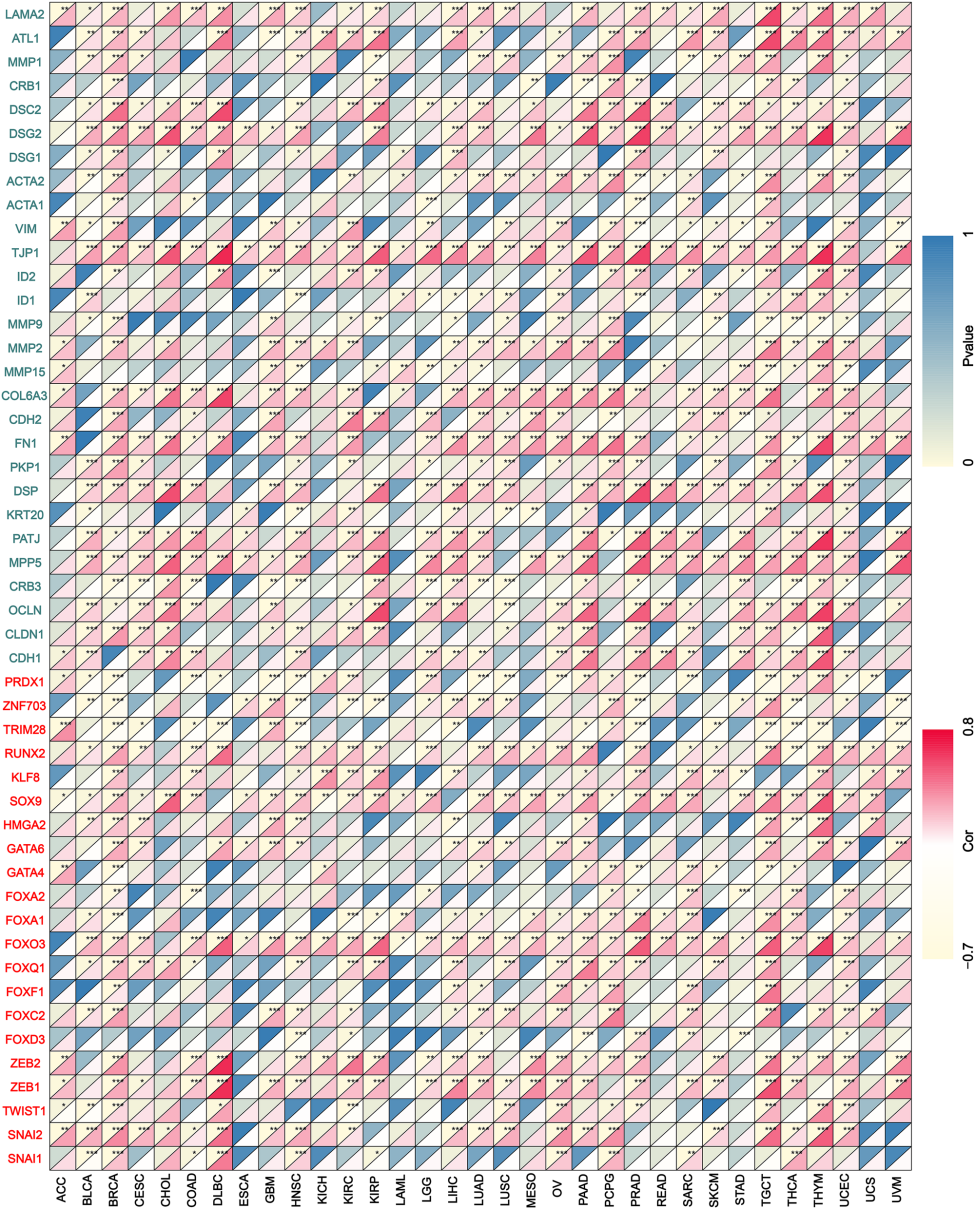
Correlation analysis of FAT1 mRNA expression with EMT marker genes. Genes marked in red are EMT transcription factors, and green genes indicate EMT transcription factor receptors.

Groundbreaking research by Ievgenia Pastushenko et al.^1^ demonstrated that FAT1 mutation promotes tumor initiation, progression, invasion, stemness, and metastasis by inducing hybrid EMT states in mouse and human skin squamous cell carcinoma. Chitrangda Srivastava et al.^41^ found that FAT1 is a novel regulator of EMT in anoxic GBM, which suggested that it may be a viable therapeutic candidate. According to Xiaoling Hu et al.^42^, FAT1 destroys the MAPK/ERK pathway and participates in the EMT process of esophageal squamous cell carcinoma. Our analysis of the correlation between FAT1 and recognized EMT transcription factors and receptors demonstrated that FAT1 is associated with EMT marker genes in almost all tumors (Fig. 7), indicating that FAT1 may be involved in the EMT process in all tumors.

Exosomes may affect tumor progression by regulating immune function, promoting tumor angiogenesis and metastasis, and interacting directly with tumor cells^43–45^. It can participate in immune response, antigen presentation, cell migration, cell differentiation, and tumor invasion. Different immune cells are also involved in tumor regulation^46^. Our results depicted that FAT1 was significantly associated with one or more exosome markers in all tumors (Fig. S2). Immune cell marker genes analysis illustrated that FAT1 was significantly associated with a variety of immune cell markers in BRCA, DLBC, GBM, HNSC, KIRC, KIRP, LIHC, LUAD, LUSC, OV, PCPG, PRAD, SARC, SKCM, STAD, TGCT, THCA, THYM, UCEC, and other tumors.

Both DNA and RNA can be methylated. The family of DNA methyltransferases play a central role in epigenetic regulation^23^. RNA methylation modification accounts for more than 60% of all RNA modifications, of which m6A is an essential methylation modification in which writers, erasers, and readers play different roles, including regulation of tissue development, circadian rhythm, DNA damage response, sex determination, T cell homeostasis and tumorigenesis^22^. The correlation between FAT1 expression and methylation marker genes is displayed in Fig. S3. Our results demonstrated a significant correlation between FAT1 and multiple methylation marker genes in different tumors except for CHOL, ESCA, LAML, and UCS.

Hypoxia has recently been confirmed to play an essential role in tumor progression^19^. Tumor hypoxia is associated with increased invasion and metastasis and shows typical driving mutation characteristics. The correlation between FAT1 expression and tumor hypoxia marker genes is revealed in Fig. S4. The findings revealed a significant correlation between FAT1 and multiple hypoxia-related mutant genes in almost all tumors except for LAML.

The dynamic equilibrium of cells, tissues, and organisms depends heavily on the autophagy pathway, which is mediated by evolutionarily conserved ATGs^24^. ATGs mutations play an important role in various diseases, including cancer^25^. The correlation between FAT1 expression and autophagy-related marker genes is illustrated in Fig. S5.

## Discussion

FAT atypical cadherin 1 (FAT1) encodes protocadherin, one of the most frequently mutated genes in human cancers. Studies conducted over the past 20 years have demonstrated that FAT1 regulates multiple signaling pathways, including Wnt/β-catenin, Hippo and MAPK/ ERK, to affect the proliferation, migration, and invasion of various tumor cells^1,47^. In this study, FAT1 was found to have a mutation rate of > 10% in more than ten tumors. It has not been established whether FAT1 plays a role in the pathogenesis of different tumors through some common molecular mechanisms; therefore, we performed a comprehensive pan-cancer analysis of FAT1 based on data in TCGA, CPTAC, and GEO from an overall tumor perspective, focusing on gene expression, gene alterations, protein phosphorylation, and prognosis.

FAT1 is highly expressed in most tumors. However, the prognostic survival analysis of the FAT1 gene data suggested different conclusions for different tumors. In the present study, we combined different prognostic analysis methods and data. Finally, we revealed that high FAT1 expression predicted a worse prognosis in all tumors except STAD, STES, ESCA, KIRP, UCS, UCEC, READ, and TGCT. This is according to certain recent studies^47^.

According to previous studies, FAT1 is mutated in a variety of tumors. This study analyzed the relationship between FAT1 mutations and patient survival in the four tumors with the highest mutation rates. We revealed that FAT1 mutations in UCEC predicted a better prognosis for tumor patients, whereas they predicted poor survival in HNSC patients. According to the OS, patients with FAT1 mutation had a better prognosis, as revealed by the mutation data from TMB and immunotherapy (MSKCC, NatGenet 2019)^13^. Furthermore, we found that FAT1 was significantly correlated with TMB in ACC, KIRC, LAML, PAAD, READ, STAD, THYM, LGG, and LUAD tumors. This result suggested that FAT1 mutations may serve as immunotherapeutic markers for these tumors and may be useful for guiding novel immunotherapies.

The PTMs regulate the function of most eukaryotic proteins^48^, and protein phosphorylation is very closely associated with tumors^49^. In this study, the database statistically predicted the most phosphorylated sites and protein kinases of FAT1 protein to explore potential associations between FAT1 and tumors. Recent studies have demonstrated that deletion of FAT1 in cutaneous squamous cell carcinoma accelerates tumor initiation, malignant progression and promotes the EMT phenotype^1^. We found a significant association between FAT1 mRNA level and EMT phenotype-related marker genes in almost all tumor types, implying that FAT1 may play a role in the EMT phenotype in other tumors. Studies analyzing the correlation between FAT1 and markers genes such as exosomal and immune, methylation, hypoxia, and autophagy have produced a number of additional useful findings. These phenomena need to be confirmed by further research.

For the first time, this study provided evidence for a potential correlation between FAT1 expression and MSI or TMB in all TCGA tumors. We also integrated information on FAT1 binding components and FAT1 expression-related genes in all tumors. We performed enrichment analyses to identify the potential effects of FAT1 in tumor pathways, reverse regulation of cell differentiation, transcription factor binding, and epithelial cell development.

FAT1 has been reported to have immunotherapeutic potential in several tumors^50,51^. We applied the immunological deconvolution method to observe the statistical correlation between FAT1 expression and the infiltration level of 22 immune cells in pan-cancer. FAT1 may play a function in the immunotherapy of THYM as a result of the highly substantial association between THYM and many immune cells in THYM. Additionally, we found that FAT1 expression was significantly and positively correlated with immune scores of BRCA, DLBC, LGG, OV, and PCPG based on the calculated stromal, immune, and ESTIMATE scores for each patient in the TCGA pan-cancer dataset of 9530 tumor samples from a total of 39 tumor types. These findings can provide research directions for tumor immunotherapy based on FAT1.

Our first pan-cancer analysis of FAT1 demonstrated a statistical correlation between FAT1 expression and clinical prognosis, protein phosphorylation, MSI, TMB, TME, and immune cell infiltration, which helped comprehend the role of FAT1 in tumorigenesis from the perspective of clinical tumor samples. Moreover, FAT1 mutations were also found to be closely associated with immunotherapy and may develop into tumor immunotherapy markers. This study also helped to broaden the research on FAT1 in tumors. Undeniably, this study was based on extensive tumor data, but it had the limitation of lacking rigorous experiments to validate the findings. More reliable experiments will be needed to validate the various potential roles of FAT1 in tumors.

## Supplementary Information

Supplementary Figure S1.

Supplementary Figure S2.

Supplementary Figure S3.

Supplementary Figure S4.

Supplementary Figure S5.

Supplementary Legends.

Supplementary Tables.

## Data Availability

These data are drawn from the following resources in the public domain. UCSC Xena (http://xena.ucsc.edu/), the cohort is TCGA Pan-Cancer (PANCAN: https://pancanatlas.xenahubs.net). All data in this study were permitted for use.
