# Supplementary figures and images for: A pan-cancer analysis of the FAT1 in human tumors
Source: Sci Rep. 2022 Dec 14;12:21598. doi: 10.1038/s41598-022-26008-1 (PMC9751142; doi:10.1038/s41598-022-26008-1)

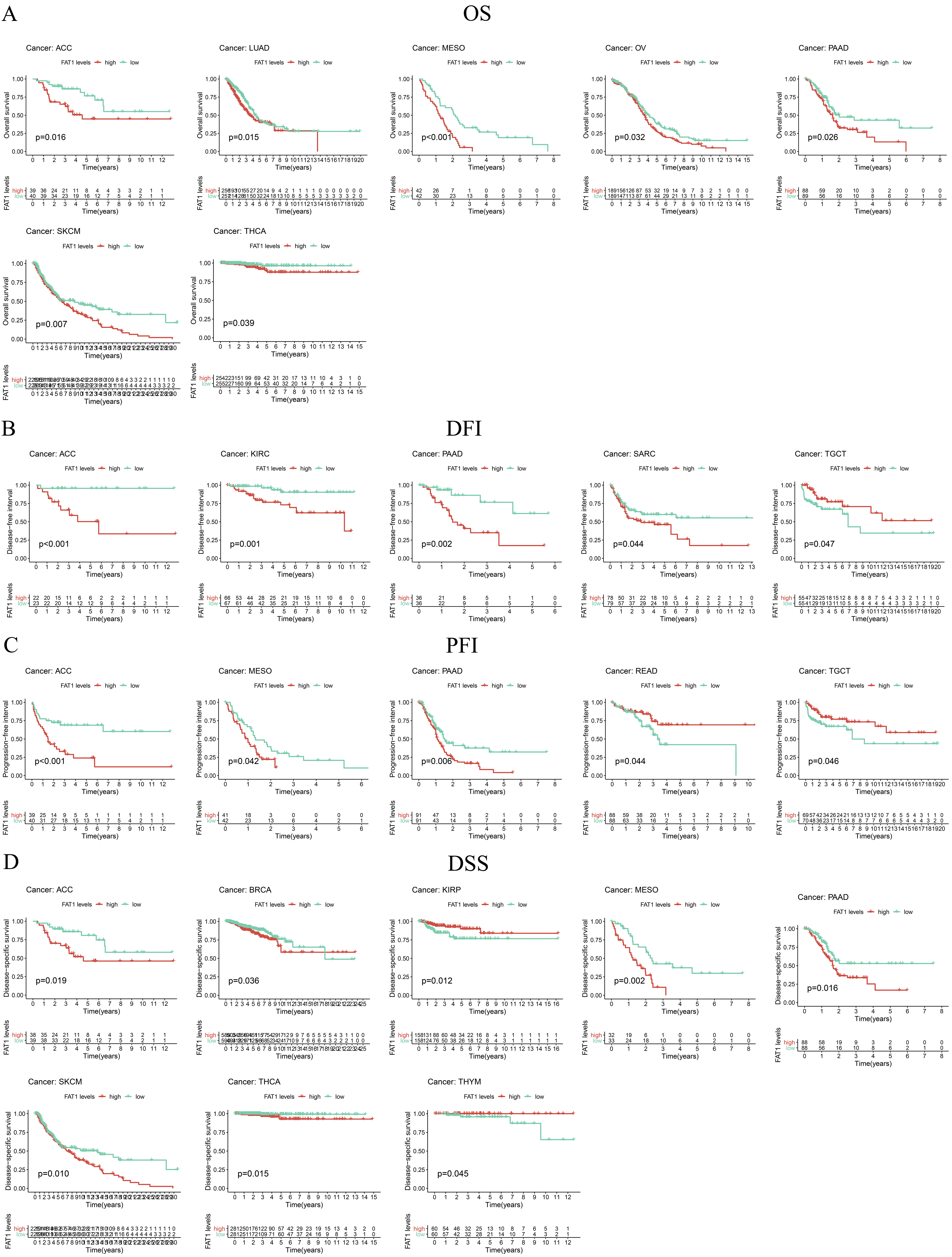

Supplement: Supplementary file 1 — Supplementary Figure S1. [file 41598_2022_26008_MOESM1_ESM.tif]

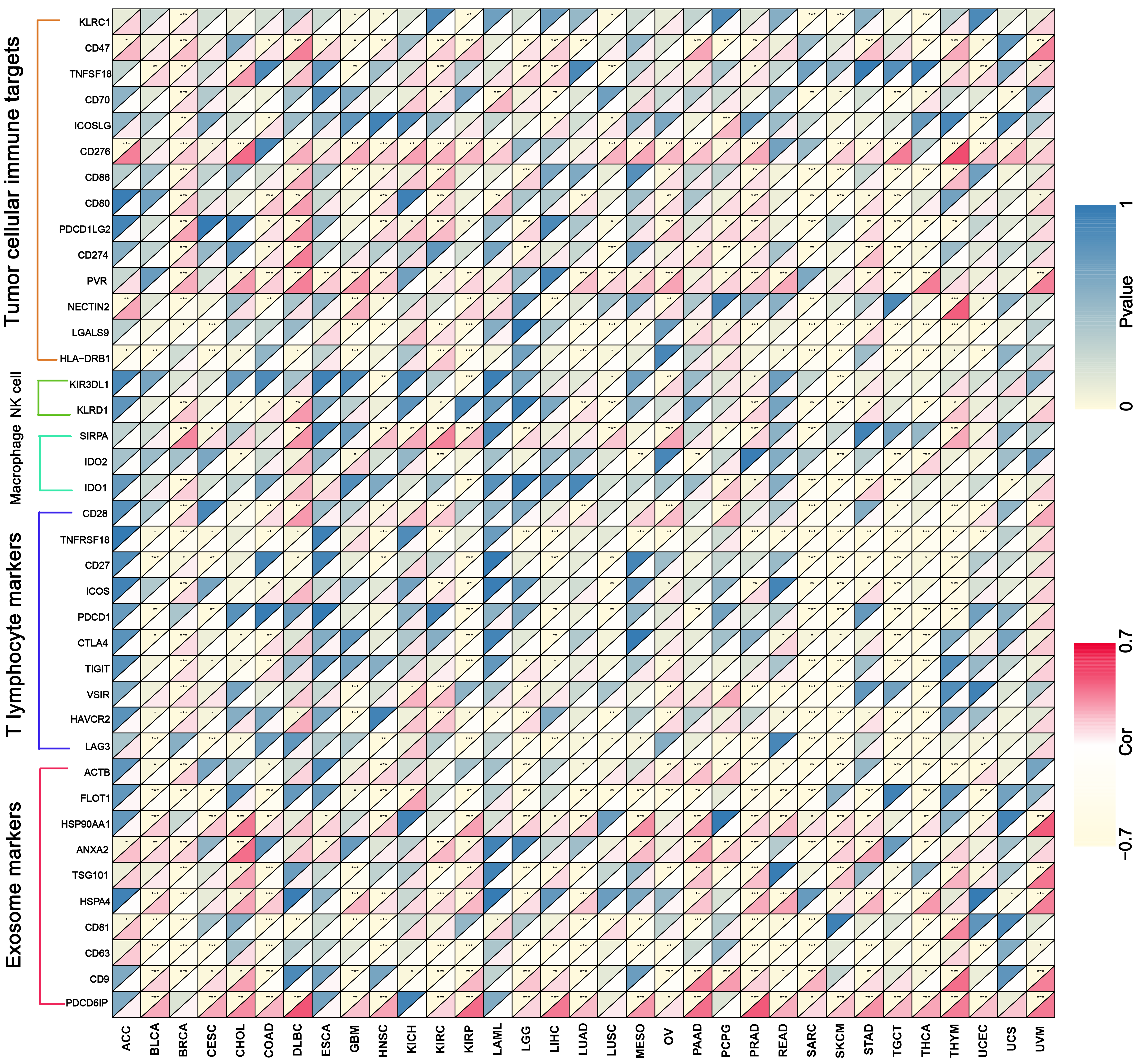

Supplement: Supplementary file 2 — Supplementary Figure S2. [file 41598_2022_26008_MOESM2_ESM.tif]

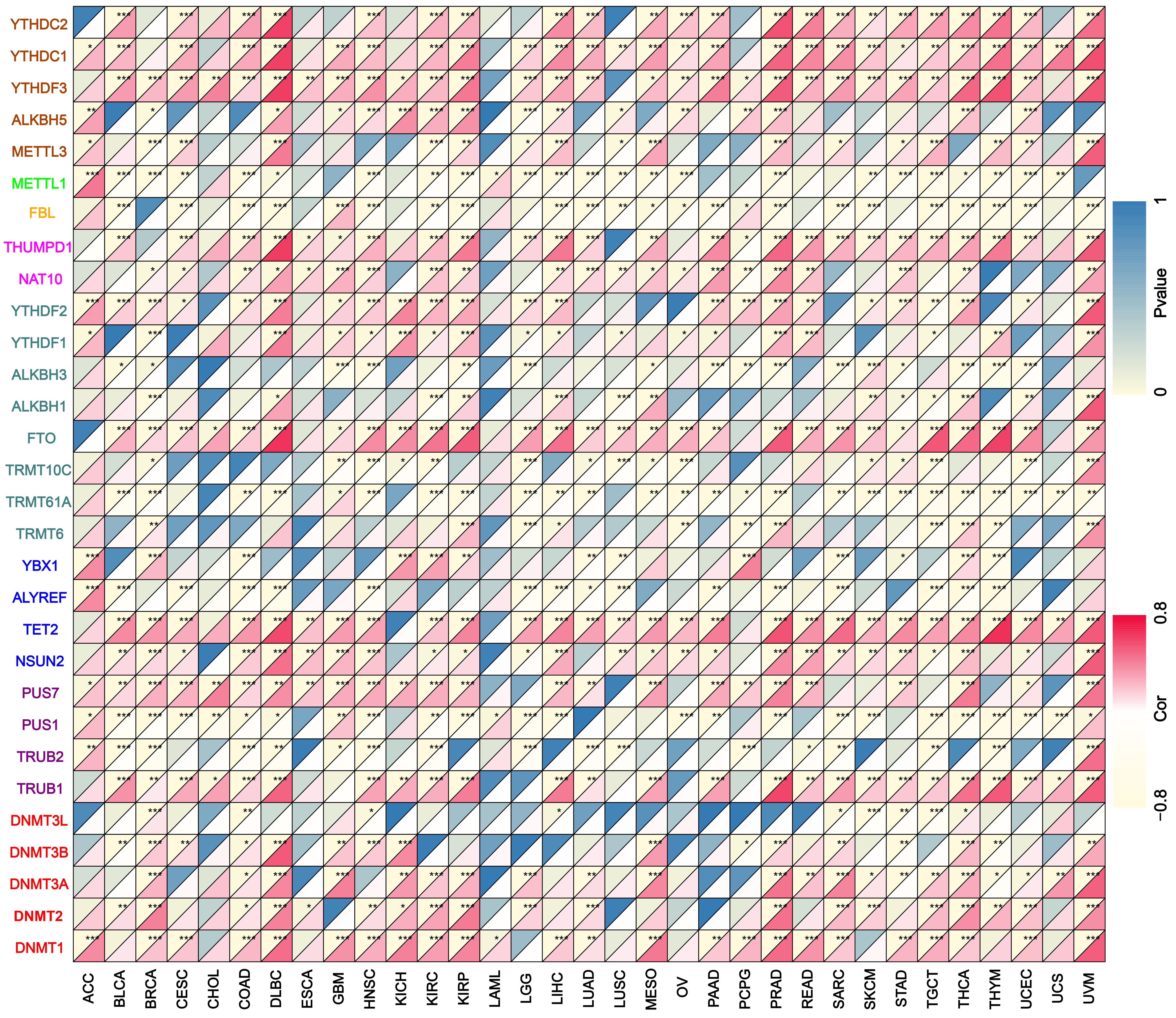

Supplement: Supplementary file 3 — Supplementary Figure S3. [file 41598_2022_26008_MOESM3_ESM.tif]

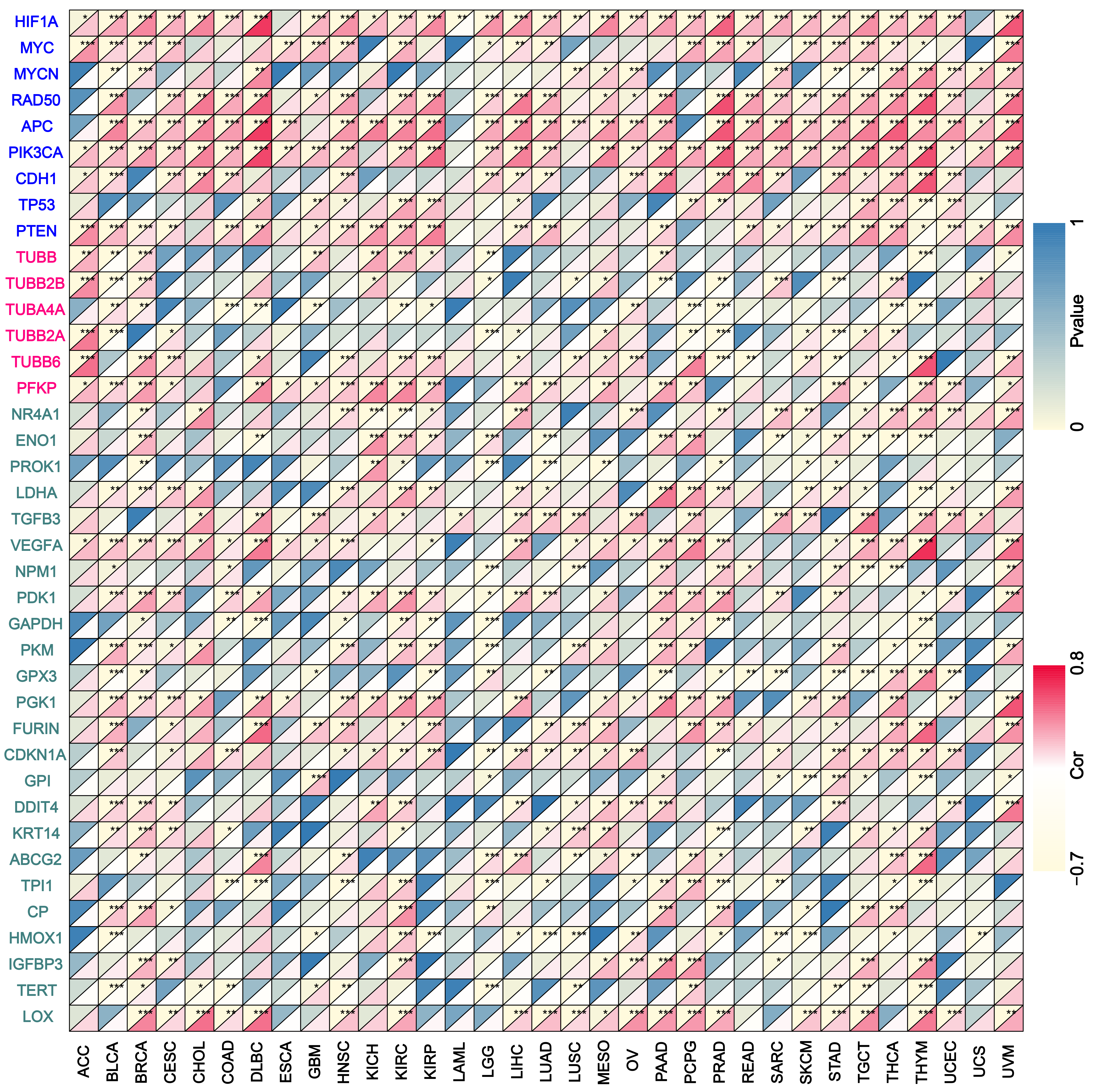

Supplement: Supplementary file 4 — Supplementary Figure S4. [file 41598_2022_26008_MOESM4_ESM.tif]

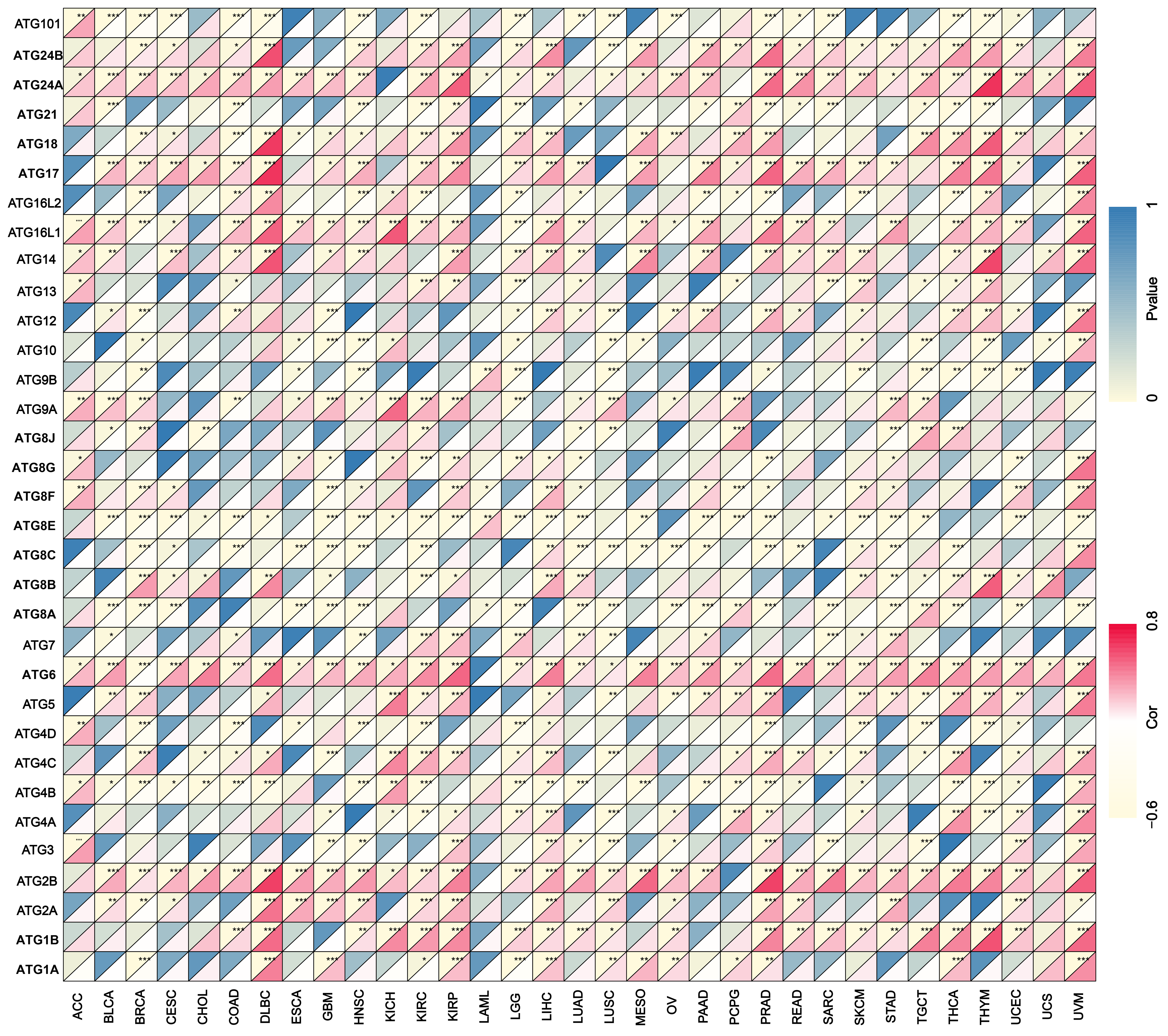

Supplement: Supplementary file 5 — Supplementary Figure S5. [file 41598_2022_26008_MOESM5_ESM.tif]
