# Supplementary material for: A pan-cancer analysis of the FAT1 in human tumors
Source: Sci Rep. 2022 Dec 14;12:21598. doi: 10.1038/s41598-022-26008-1 (PMC9751142; doi:10.1038/s41598-022-26008-1)
Supplement: Supplementary file 6 — Supplementary Legends. [file 41598_2022_26008_MOESM6_ESM.docx]

**Fig. S1. Kaplan-Meier method to analyze the prognostic value of FAT1 in pan-cancer.**

(**A**) Overall survival. (**B**) Disease-free interval. (**C**) Progression-free interval. (**D**) Disease-specific survival.

**Fig. S2. Correlation analysis of FAT1 mRNA expression with exosome and tumor immune marker genes.**

**Fig. S3. Correlation analysis of FAT1 mRNA expression with methylation (methyltransferase, Ψ, ac4 C, m1 A, m5 C, m6 A, m7 G, Nm) marker genes.**

Ψ, pseudouridine; ac4 C, N4 -acetylcytidine; m1A, N1 -methyladenosine; m5C, 5-methylcytidine; m6A, N6 -methyladenosine; m7G, N7 -methylguanosine; Nm, ribose methylation.

**Fig. S4. Correlation analysis of FAT1 mRNA expression with tumor hypoxia marker genes.**

Blue are significantly mutated genes associated with hypoxia; red are genes significantly associated with hypoxia in various tumors, and green represents HIF1A targets.

**Fig. S5. Correlation analysis of FAT1 mRNA expression with autophagy marker genes.**
