# Supplementary material for: A pan-cancer analysis of the FAT1 in human tumors
Source: Sci Rep. 2022 Dec 14;12:21598. doi: 10.1038/s41598-022-26008-1 (PMC9751142; doi:10.1038/s41598-022-26008-1)
Supplement: Supplementary file 7 — Supplementary Tables. [file 41598_2022_26008_MOESM7_ESM.docx]

Table S1. Predicted FAT1 protein phosphorylation.

| **Phosphosites** | **P-site Match（Sequence）** | | | | | | | | | | | | | | | | **Protein Kinase Match（Top 1）** | | |
| --- | --- | --- | --- | --- | --- | --- | --- | --- | --- | --- | --- | --- | --- | --- | --- | --- | --- | --- | --- |
|  | **-7** | **-6** | **-5** | **-4** | **-3** | **-2** | **-1** | **0** | **1** | **2** | **3** | **4** | **5** | **6** | **7** | **Human Kinase Short Name** | | **Human Kinase Full Name** | **Kinase Predictor V2 Score** |
| S21 | L | F | Q | H | F | G | D | **S** | D | G | S | Q | R | L | E | CK2a1 (CSNK2A1) | | Casein kinase II, alpha chain | 302 |
| Y4234 | T | A | F | L | Q | R | P | **Y** | F | D | S | K | L | N | K | BTK | | Tyrosine-protein kinase BTK | 400 |
| S4237 | L | Q | R | P | Y | F | D | **S** | K | L | N | K | N | I | Y | Pim3 (AL549548) | | Threonine-Protein Kinase Pim-3 | 395 |
| S4257 | Q | V | P | V | R | P | I | **S** | Y | T | P | S | I | P | S | MAPKAPK3 | | Mitogen activated protein kinase activated protein kinase-3 | 527 |
| Y4258 | V | P | V | R | P | I | S | **Y** | T | P | S | I | P | S | D | BTK | | Tyrosine-protein kinase BTK | 335 |
| T4259 | P | V | R | P | I | S | Y | **T** | P | S | I | P | S | D | S | JNK1 (MAPK8) | | Mitogen-activated protein kinase 8 | 360 |
| S4261 | R | P | I | S | Y | T | P | **S** | I | P | S | D | S | R | N | GSK3A | | Glycogen synthase kinase-3 alpha | 391 |
| S4264 | S | Y | T | P | S | I | P | **S** | D | S | R | N | N | L | D | CDK7 | | Cyclin-dependent kinase 7 | 311 |
| S4266 | T | P | S | I | P | S | D | **S** | R | N | N | L | D | R | N | HSER (GUCY2C) | | Heat-stable enterotoxin receptor | 312 |
| T4288 | P | E | H | P | E | F | S | **T** | F | N | P | E | S | V | H | Pim3 (AL549548) | | Threonine-Protein Kinase Pim-3 | 169 |
| S4293 | F | S | T | F | N | P | E | **S** | V | H | G | H | R | K | A | CDK7 | | Cyclin-dependent kinase 7 | 316 |
| S4320 | P | P | P | S | N | S | P | **S** | D | S | D | S | I | Q | K | GSK3B | | Glycogen synthase kinase-3 beta | 416 |
| S4324 | N | S | P | S | D | S | D | **S** | I | Q | K | P | S | W | D | ERK1 | | Mitogen-activated protein kinase 3 | 410 |
| Y4334 | K | P | S | W | D | F | D | **Y** | D | T | K | V | V | D | L | FGFR1 | | Basic fibroblast growth factor receptor 1 | 549 |
| S4346 | V | D | L | D | P | C | L | **S** | K | K | P | L | E | E | K | MAPKAPK2 | | MAP kinase-activated protein kinase 2 | 355 |
| S4355 | K | P | L | E | E | K | P | **S** | Q | P | Y | S | A | R | E | ATR | | Serine-protein kinase ATR | 575 |
| S4359 | E | K | P | S | Q | P | Y | **S** | A | R | E | S | L | S | E | ERK1 | | Mitogen-activated protein kinase 3 | 465 |
| S4363 | Q | P | Y | S | A | R | E | **S** | L | S | E | V | Q | S | L | PKACa (PRKACA) | | cAMP-dependent protein kinase, alpha-catalytic subunit | 481 |
| S4365 | Y | S | A | R | E | S | L | **S** | E | V | Q | S | L | S | S | p38a MAPK (MAPK14) | | Mitogen-activated protein kinase 14 | 376 |
| S4369 | E | S | L | S | E | V | Q | **S** | L | S | S | F | Q | S | E | CK1a1 (CSNK1A1) | | Casein kinase I, alpha isoform | 424 |
| S4371 | L | S | E | V | Q | S | L | **S** | S | F | Q | S | E | S | C | p38d MAPK (MAPK13) | | Mitogen-activated protein kinase 13 | 360 |
| S4372 | S | E | V | Q | S | L | S | **S** | F | Q | S | E | S | C | D | Pim3 (AL549548) | | Threonine-Protein Kinase Pim-3 | 382 |
| S4375 | Q | S | L | S | S | F | Q | **S** | E | S | C | D | D | N | G | CK1a1 (CSNK1A1) | | Casein kinase I, alpha isoform | 377 |
| S4377 | L | S | S | F | Q | S | E | **S** | C | D | D | N | G | Y | H | CDK10 | | Cell division protein kinase 10 | 314 |
| S4393 | D | T | S | D | W | M | P | **S** | V | P | L | P | D | I | Q | GSK3A | | Glycogen synthase kinase-3 alpha | 328 |
| Y4405 | D | I | Q | E | F | P | N | **Y** | E | V | I | D | E | Q | T | SYK | | Tyrosine-protein kinase SYK | 518 |
| T4412 | Y | E | V | I | D | E | Q | **T** | P | L | Y | S | A | D | P | ERK5 (MAPK7) | | Mitogen-activated protein kinase 7 | 268 |
| Y4415 | I | D | E | Q | T | P | L | **Y** | S | A | D | P | N | A | I | FRK | | Fyn-related kinase | 380 |
| T4424 | A | D | P | N | A | I | D | **T** | D | Y | Y | P | G | G | Y | mTOR/FRAP | | Mechanistic Target Of Rapamycin Kinase | 170 |
| Y4426 | P | N | A | I | D | T | D | **Y** | Y | P | G | G | Y | D | I | AXL | | AXL oncogene-encoded protein-tyrosine kinase UFO | 572 |
| Y4427 | N | A | I | D | T | D | Y | **Y** | P | G | G | Y | D | I | E | TEC | | Tyrosine-protein kinase Tec | 460 |
| S4435 | P | G | G | Y | D | I | E | **S** | D | F | P | P | P | P | E | NEK10 | | Serine-threonine-protein kinase Nek10 | 346 |
| S4458 | P | P | L | P | P | E | F | **S** | N | Q | F | E | S | I | H | MAPKAPK2 | | MAP kinase-activated protein kinase 2 | 324 |
| S4480 | A | G | S | L | G | S | S | **S** | R | N | R | Q | R | F | N | PKCt (PRKCQ) | | Protein kinase C, theta type | 308 |
| Y4496 | N | Q | Y | L | P | N | F | **Y** | P | L | D | M | S | E | P | CSK | | Tyrosine-protein kinase CSK | 320 |
| S4501 | N | F | Y | P | L | D | M | **S** | E | P | Q | T | K | G | T | mTOR/FRAP | | Mechanistic Target Of Rapamycin Kinase | 291 |
| T4505 | L | D | M | S | E | P | Q | **T** | K | G | T | G | E | N | S | ERK1 | | Mitogen-activated protein kinase 3 | 117 |
| S4512 | T | K | G | T | G | E | N | **S** | T | C | R | E | P | H | A | PIM1 | | Proto-oncogene serine-threonine-protein kinase Pim-1 | 481 |
| T4513 | K | G | T | G | E | N | S | **T** | C | R | E | P | H | A | P | Pim3 (AL549548) | | Threonine-Protein Kinase Pim-3 | 135 |
| Y4542 | E | S | M | P | M | S | V | **Y** | A | S | T | A | S | C | S | SRM (SRMS) | | Tyrosine-protein kinase Srms | 332 |
| S4564 | V | E | S | E | V | M | M | **S** | D | Y | E | S | G | D | D | CK2a2 (CSNK2A2) | | Casein kinase II, alpha' chain | 450 |
| Y4566 | S | E | V | M | M | S | D | **Y** | E | S | G | D | D | G | H | SRM (SRMS) | | Tyrosine-protein kinase Srms | 470 |
| S4568 | V | M | M | S | D | Y | E | **S** | G | D | D | G | H | F | E | IKKa (CHUK) | | Inhibitor of nuclear factor kappa-B kinase alpha subunit | 335 |
| T4578 | D | G | H | F | E | E | V | **T** | I | P | P | L | D | S | Q | BLK | | Tyrosine-protein kinase BLK | 126 |
| S4584 | V | T | I | P | P | L | D | **S** | Q | Q | H | T | E | V | _ | ATR | | Serine-protein kinase ATR | 577 |
